# Images in Vascular Medicine: Acute peripheral artery occlusion and ischemic stroke in a patient with COVID-19

**DOI:** 10.1177/1358863X20945020

**Published:** 2020-08-19

**Authors:** Antonia Nayanne de Almeida Lima, Mariana Santos Leite Pessoa, Carla Franco Costa Lima, Pablo Picasso de Araújo Coimbra, Jorge Luis Bezerra Holanda

**Affiliations:** Radiology Department, Fortaleza General Hospital, Fortaleza-CE, Brazil

A 46-year-old man with hypertension and diabetes was admitted to the emergency department with one day of left-sided weakness, mainly in the lower limb. He additionally reported pain and weakness in the right hand, with impaired wrist and finger movements, as well as bluish discoloration and coolness to touch. The patient reported a dry cough and headache associated with anosmia for about 1 week.

Upon physical examination, the patient was in no acute distress, hypertensive (160/70 mmHg), tachycardic (137 bpm), and eupneic (respiration rate 20; SaO_2_ 99% on room air). Facial inspection demonstrated slight erasure of the nasolabial groove on the left, as well as difficulty in keeping the left eye closed, and loss of muscle strength in the left upper and lower limbs. The right limb demonstrated cyanotic and cool fingers with distal necrosis of the fourth right finger. The right radial pulse was not palpable; there was slow capillary filling time in the right hand.

Laboratory testing was notable for a positive antigen nasal swab for SARS-CoV-2; C-reactive protein: 220 mg/dL; D-dimer: 700 ng/mL; and fibrinogen: 430 mg/dL.

Chest computed tomography (CT) demonstrated sparse ground-glass opacities in both lungs, notably in the periphery (Panel A: arrows), consistent with COVID-19 pneumonia.

**Panel A.**
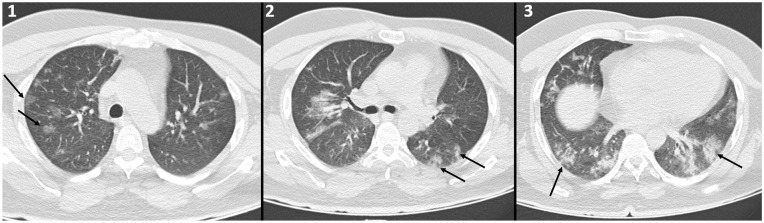


A non-contrast head CT demonstrated a cortico-subcortical hypodensity in the right temporoparietal region (Panel B: arrows), in the territory of the right middle cerebral artery, compatible with acute/subacute ischemic stroke.

**Panel B.**
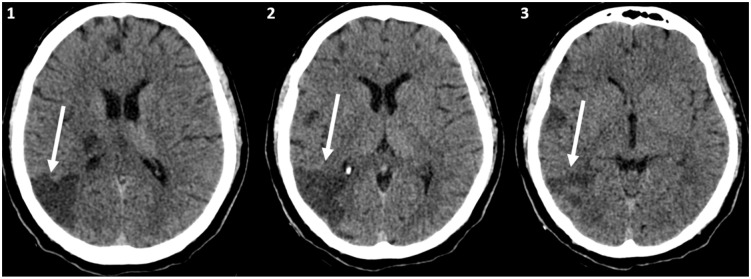


CT angiography of the right upper limb showed a filling defect in the distal brachial artery (Panel C: arrows), as well as the radial and ulnar arteries, with no filling in the distal segments (Panels D-1 and D-2, respectively: arrows).

**Panel C.**
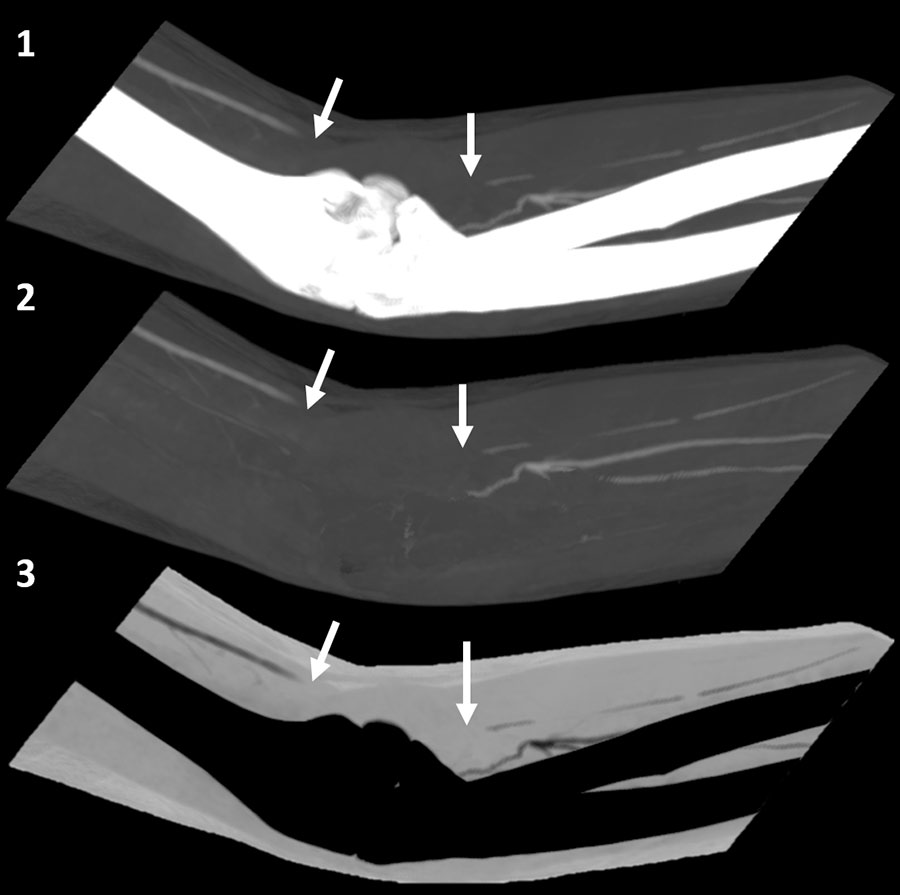


**Panel D.**
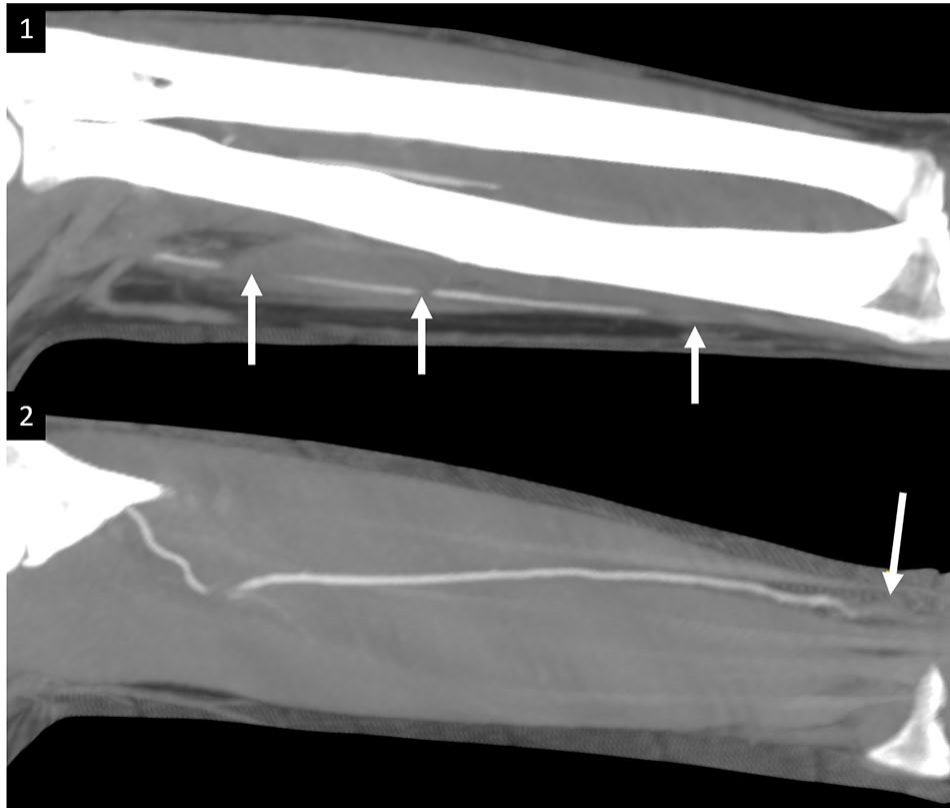


Therapeutic anticoagulation and limb heating were initiated to limit ischemic injury as advised by our vascular surgery colleagues. Antibiotics and corticosteroids were additionally added. The patient stabilized over the course of his hospital stay and was discharged with outpatient follow-up.

Abnormal coagulation parameters have been reported in hospitalized patients with severe coronavirus disease (COVID-19)^1,2^ and elevated D-dimer levels have been associated with increased mortality.^3^ The case reported shows a young patient, with no history of previous coagulopathy, who presented with acute cerebral ischemia and artery occlusion of the right arm associated with COVID-19, with important clinical repercussions, corroborating the prothrombotic effect associated with the SARS-CoV-2 infection.

‘Images in vascular medicine’ is a regular feature of *Vascular Medicine*. Readers may submit original, unpublished images related to clinical vascular medicine. Submissions may be sent to: Heather Gornik, Editor in Chief, *Vascular Medicine*, via the web-based submission system at http://mc.manuscriptcentral.com/vascular-medicine
